# Maternal Vaccination Coverage in Brazil From 2018 to 2022: A Cross-Sectional Descriptive Analysis Using Minimum and Maximum Estimates

**DOI:** 10.7759/cureus.93736

**Published:** 2025-10-02

**Authors:** Carolina Barbosa Carvalho do Carmo, Luiza de Lima Pereira

**Affiliations:** 1 Medicine and Health School, Catholic University of Brasília, Brasília, BRA

**Keywords:** immunization programs, maternal health services, pregnant women, vaccination coverage, vaccines‎

## Abstract

Background: Brazil has historically achieved high immunization coverage through its National Immunization Program (PNI). Vaccination during pregnancy is essential to protect both mothers and infants, yet routine immunization services were disrupted during the COVID-19 pandemic. Monitoring maternal vaccination coverage is crucial to inform public health strategies.

Methods: This cross-sectional descriptive study analyzed publicly available data from the Department of Information and Informatics of the Unified Health System (DATASUS) for 2018-2022. Pregnant women aged 18-49 years were included. Vaccination coverage was estimated using two scenarios: minimum coverage (two doses: influenza and Tdap (tetanus, diphtheria, and acellular pertussis)) and maximum coverage (five doses: influenza, Tdap, and three doses of hepatitis B). Coverage was calculated as the ratio of doses administered to expected doses, using live births as the denominator.

Results: Minimum coverage increased in all regions, ranging from 35.7-43.0% in 2018 to 44.5-86.6% in 2022. Maximum coverage ranged from 14.3-17.2% in 2018 to 17.8-34.6% in 2022. The North region showed the largest relative gain in minimum coverage (+43.4 percentage points), while the South and Southeast regions experienced temporary declines in 2021 compared to 2020 (-12 to -13 points), followed by a recovery in 2022.

Conclusions: Maternal vaccination coverage in Brazil demonstrated resilience after the initial decline during the COVID-19 pandemic, with minimum coverage exceeding 70% in the North, Northeast, and Midwest regions by 2020, while the South and Southeast regions remained below this threshold. Regional heterogeneity, particularly lower recovery in the Southeast, highlights the need for tailored strategies to sustain and expand maternal immunization. These findings provide evidence to guide public health interventions and reinforce the importance of continuous monitoring of maternal vaccination.

## Introduction

Brazil has long been recognized as a global reference in immunization, anchored by a robust National Immunization Program (PNI) that provides vaccines free of charge and has achieved major control or elimination of vaccine-preventable diseases over the past five decades [1]. This longstanding investment in publicly funded vaccination infrastructure underpins high programmatic capacity and nationwide reach [1].

Vaccination during pregnancy is a proven strategy to protect both the mother and the newborn, with passive transplacental transfer of IgG conferring early infant protection [2,3]. In Brazil, current national recommendations for pregnant women include annual influenza vaccination, a single Tdap (tetanus, diphtheria, and acellular pertussis) dose during pregnancy (from the 20th gestational week), and completion of the three-dose hepatitis B series for those without prior immunization [4,5]. These recommendations reflect the dual goal of reducing maternal morbidity and providing early-life protection for infants [2,3].

The COVID-19 pandemic disrupted routine health services globally, including Brazil, contributing to declines in routine immunization coverage in 2020 and subsequent years, with heterogeneous regional patterns [6,7]. Monitoring trends in maternal immunization is therefore essential to prevent gaps in protection and to guide targeted interventions within the Brazilian context [6,7].

The Department of Information and Informatics of the Unified Health System (DATASUS) is responsible for developing and maintaining national health information systems, as well as the interoperability infrastructure via the National Health Data Network (RNDS). Vaccine doses administered are recorded by public and integrated private facilities (public and integrated private sites) in the National Immunization Program Information System (SIPNI/new SIPNI), which is part of the PNI [8-10], or in local systems connected to the RNDS. These are administrative records of doses and do not correspond to the "compulsory notification" system, which applies only to notifiable diseases and feeds the Information System for Notifiable Diseases (SINAN) database [11,12]. For well-established campaign vaccines such as influenza, reporting continues through the new SIPNI [13,14]. Recent operational guidelines emphasize that each dose must be recorded only once in a single system to avoid duplication [13,15]. Therefore, the indicators used in this study are based on doses entered into the system (administrative data) rather than individual-level vaccination status, which may capture programmatic variations (campaign timing, extended schedules, delayed data entry) in addition to real changes in coverage [16,17].

The objective of this study was to analyze vaccination coverage among pregnant women in Brazil from 2018 to 2022 using adjusted minimum and maximum estimates that account for variability in the hepatitis B schedule, thereby providing evidence relevant to maternal-infant health and public health decision-making.

## Materials and methods

This was a cross-sectional descriptive study using publicly available secondary data from the Brazilian Ministry of Health database (DATASUS) for the years 2018 to 2022. The study population included pregnant women aged 18 to 49 years.

Variables analyzed

The variables extracted were the total number of doses administered to pregnant women, the number of live births, and adjusted estimates of vaccination coverage (minimum and maximum). All vaccines administered during pregnancy were considered together, as the dataset does not allow stratification by type.

Rationale for dose estimation

Brazilian prenatal guidelines recommend one dose of influenza vaccine, one dose of Tdap, and three doses of hepatitis B vaccine. The hepatitis B schedule, however, varies depending on previous immunization history. Women who had already completed the three-dose schedule before or during a previous pregnancy may not receive any new doses, while those who are partially immunized would only complete the series, and women who have never been vaccinated would receive all three doses. To account for this variability, vaccination coverage was calculated under two scenarios: (i) minimum coverage, assuming two doses per pregnant woman (influenza and Tdap, with prior completion of hepatitis B), and (ii) maximum coverage, assuming five doses per pregnant woman (influenza, Tdap, and the full three-dose hepatitis B series).

Calculations

Vaccination coverage was estimated using the following formula:

$Coverage(\%) = \frac{DA}{LB \times EDP} \times 100$

where DA is the number of doses administered, LB is the number of live births, and EDP is the number of doses expected per pregnant woman.

This formula was applied for both the minimum (two doses) and maximum (five doses) scenarios in each year and region, producing an interval of coverage estimates.

## Results

When analyzing minimum coverage, all five regions demonstrated progressive improvement between 2018 and 2022. The Northeast region reached the highest minimum coverage by 2022 (86.6%), while the North region started at a lower level in 2018 (36.8%) but showed substantial growth, achieving 80.2% in 2022. The Midwest and South regions reached 77.2% and 60.5%, respectively, while the Southeast region remained the lowest (44.5%).

In terms of maximum coverage, the Northeast and Midwest regions presented the highest estimates, peaking at 34.6% and 30.9%, respectively, in 2022. The North region exhibited a low baseline in 2018 (14.7%) but more than doubled by 2022 (32.1%). The South and Southeast regions showed intermediate results, starting at approximately 14-15% in 2018 and reaching 17.8-24.2% in 2022. The North region also had the largest relative increase in minimum coverage (+43.4 percentage points). The South region experienced temporary reductions of 12-13 percentage points in 2021 compared to 2020, but recovered in 2022.

**Figure 1:**
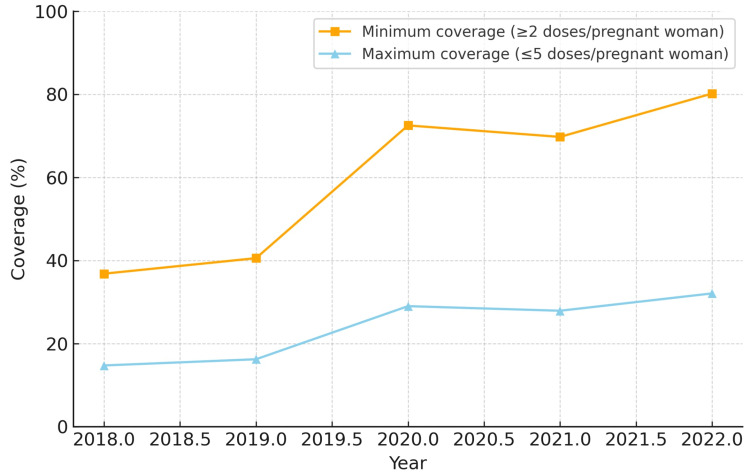
Evolution of maternal vaccination coverage in the North region of Brazil (2018-2022)

**Figure 2:**
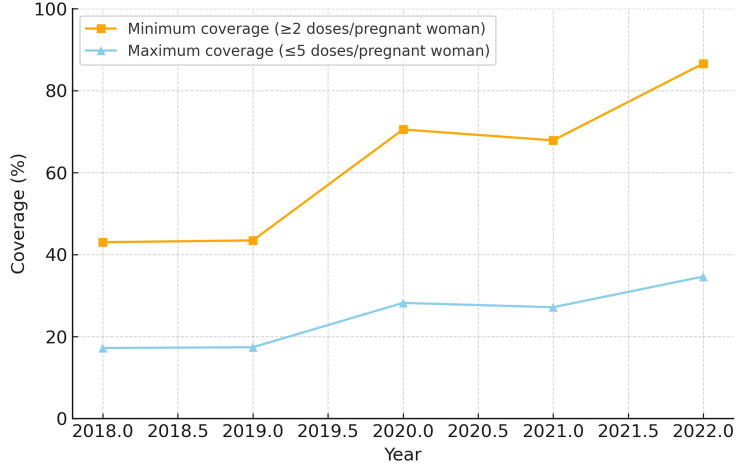
Evolution of maternal vaccination coverage in the Northeast region of Brazil (2018-2022)

**Figure 3:**
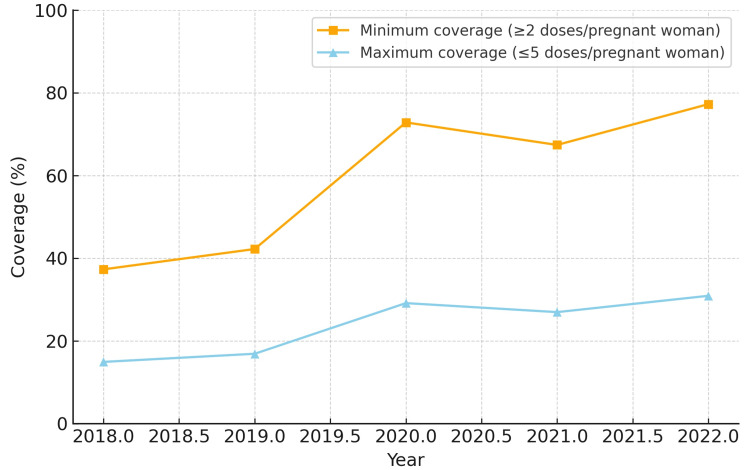
Evolution of maternal vaccination coverage in the Midwest region of Brazil (2018-2022)

**Figure 4:**
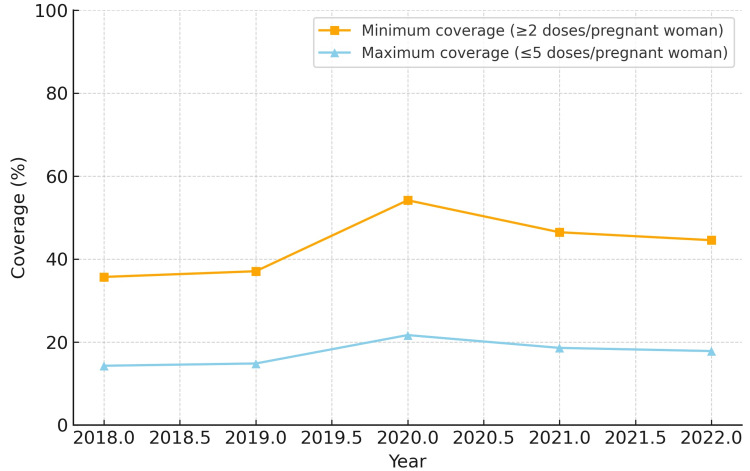
Evolution of maternal vaccination coverage in the Southeast region of Brazil (2018-2022)

**Figure 5:**
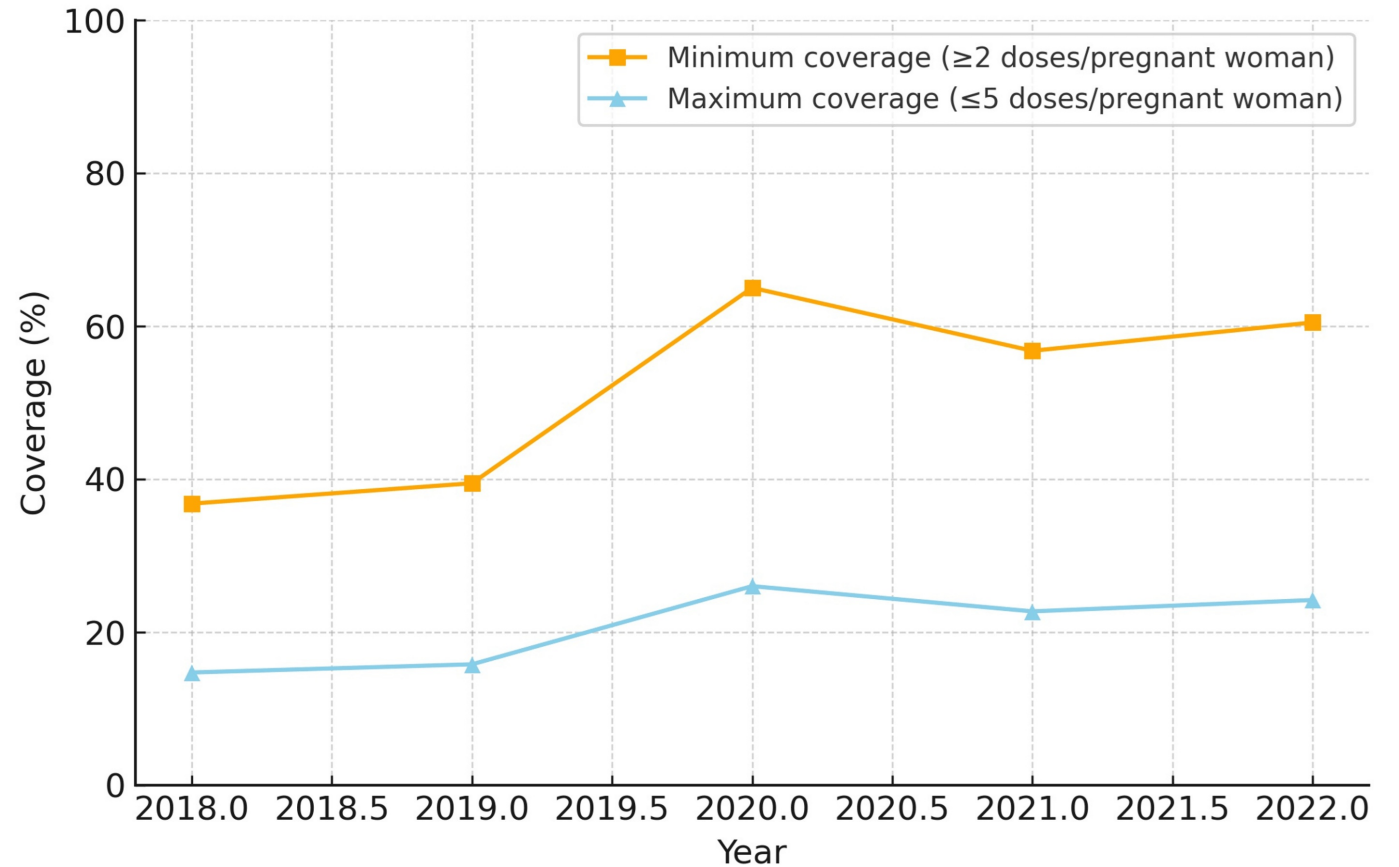
Evolution of maternal vaccination coverage in the South region of Brazil (2018-2022)

**Figure 6:**
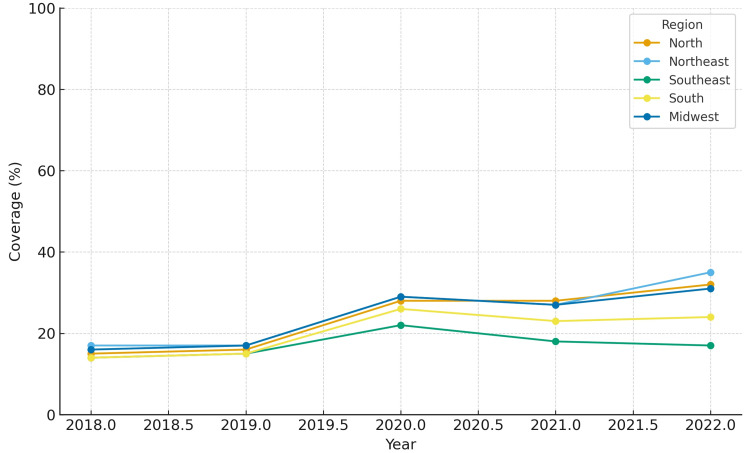
Comparative levels of maximum maternal vaccination coverage across regions (2018-2022)

**Figure 7:**
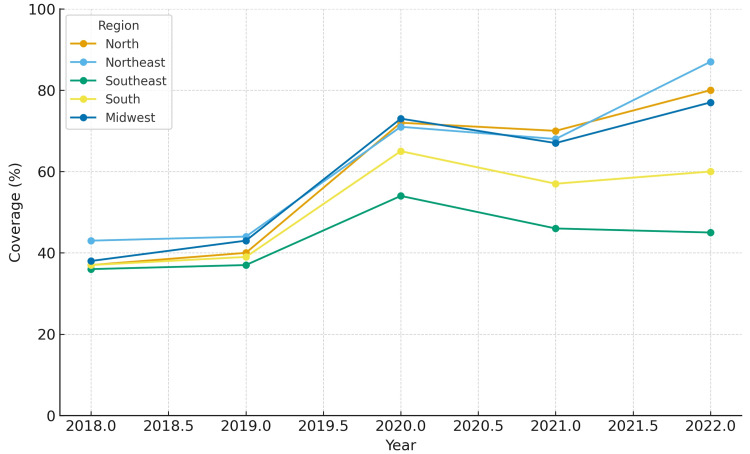
Comparative levels of minimum maternal vaccination coverage across regions (2018-2022)

The coverage for each region from 2018 to 2022 is depicted in Figures 1-5. Comparative levels across regions are depicted in Figures 6-7. Table 1 provides a summary of the initial, final, and average values for minimum and maximum coverage in each region.

**Table 1:** Summary of maternal vaccination coverage by region in Brazil (2018-2022) Min: Minimum; Max: Maximum; Avg: Average

Region	Min coverage 2018 (%)	Min coverage 2022 (%)	Min coverage Avg (%)	Max coverage 2018 (%)	Max coverage 2022 (%)	Max coverage Avg (%)
North	36.8	80.2	60.0	14.7	32.1	24.0
Northeast	43.0	86.6	62.3	17.2	34.6	24.9
Southeast	35.7	44.5	43.6	14.3	17.8	17.4
South	36.8	60.5	51.7	14.7	24.2	20.7
Midwest	37.3	77.2	59.4	14.9	30.9	23.8

## Discussion

Our findings confirm a marked reduction in maternal immunization doses in 2020 across all Brazilian regions, coinciding with the onset of the COVID-19 pandemic [18,19]. This decline has been attributed to restrictions on health services, fear of exposure, and disruptions in supply chains and staffing. Importantly, our analysis also highlights signs of a relatively rapid recovery from 2021 onwards, particularly in the North and Northeast regions [10,18]. This recovery reflects targeted catch-up strategies implemented by the Ministry of Health and supported by Pan American Health Organization (PAHO), such as extended vaccination campaigns, multivaccination drives, and active search strategies in primary care [10].

Interestingly, an unexpected surge in maternal doses between 2019 and 2020 was seen. This sharp rise is consistent with the influenza campaign in 2020, during which a governmental program aimed to advance the campaign by one month, extend it for several weeks beyond its usual window, and explicitly prioritize pregnant women [20]. These programmatic decisions likely explain the observed boom in doses administered despite the overall disruptions caused by the pandemic.

While the overall national trajectory suggests resilience, regional heterogeneity was striking. The Northeast region demonstrated a strong rebound after 2020, while the Southeast region showed a continued downward trajectory into 2021-2022. This divergence is concerning as the Southeast region concentrates the largest number of births and has historically shown higher baseline coverage [21,22].

Several hypotheses can explain the lack of recovery in the Southeast region. One possibility is the impact of vaccine hesitancy. Recent studies have suggested associations between political attitudes, misinformation, and COVID-19 vaccine uptake in Brazil [23]. Such factors may also have influenced maternal immunization confidence in certain regions. Another explanation is that programmatic national catch-up initiatives after 2021 explicitly prioritized vulnerable populations and regions with historically low coverage, such as the North and Northeast [23]. As a result, targeted investments may have accelerated recovery in these areas, while the Southeast region, with its large urban centers and higher private-sector participation, may have experienced weaker mobilization in public-sector vaccination efforts.

These findings reinforce the importance of monitoring not only national averages but also regional disparities in maternal immunization. The persistence of gaps in the Southeast region highlights the need for tailored strategies to address both demand-side barriers, such as misinformation and hesitancy, and supply-side challenges, such as organization of prenatal services and integration of private-sector reporting.

Finally, while the recovery after 2020 is encouraging, sustaining and expanding maternal vaccination coverage remains a challenge. Addressing vaccine hesitancy, strengthening prenatal care integration, and ensuring robust monitoring systems will be crucial to prevent widening inequities across Brazilian regions.

Our analysis also underlines the methodological limitation of using "doses administered" as the numerator and "live births" as the denominator. Because pregnant women may receive between two and five doses during gestation (influenza, Tdap, and up to three doses of hepatitis B, depending on prior status), coverage values must be interpreted as dose-based indicators rather than individual vaccination rates. We therefore presented both minimum (≥2 doses) and maximum (≤5 doses) expected coverage ranges to contextualize our findings.

Our study has some limitations that should be acknowledged. Firstly, the denominator used to calculate coverage was the number of live births, which does not include women who experienced late miscarriages or stillbirths. These women often attend antenatal care and receive recommended vaccines, but they are not captured in the "live births" denominator, which could theoretically overestimate vaccination coverage. Conversely, the SIPNI system restricts the age of pregnant women to 18-49 years; therefore, younger or older mothers are not included among vaccine doses administered, although their children are included among live births. This could lead to a potential underestimation of coverage. In practice, both groups represent a minority - most pregnancies occur in women aged 18-49 years and most pregnancies result in live births - so these biases may partially offset each other.

Secondly, the numerator is based on administrative data of doses entered in SIPNI/RNDS rather than individual vaccination records. This type of data is subject to delays in entry, underreporting, and potential duplication, which may affect the accuracy of the coverage estimates. Despite these limitations, the overall trends observed remain robust and consistent with programmatic changes reported by the Ministry of Health.

It is also important to note that minimum vaccination coverage does not necessarily indicate incomplete immunization, since Brazilian guidelines consider individual hepatitis B status. Thus, both minimum and maximum estimates should be interpreted as complementary scenarios, not as measures of programmatic failure.

## Conclusions

Maternal vaccination coverage in Brazil increased substantially between 2018 and 2022 when analyzed through minimum and maximum estimates, despite temporary declines associated with the COVID-19 pandemic. All regions showed improvements, especially from 2020 onwards, although regional disparities persisted, particularly in the Southeast. These findings underscore the resilience of the PNI and the need for targeted strategies to sustain and expand maternal immunization, ensuring equitable protection for mothers and infants across the country.
